# Describing Caregiver and Clinician Experiences with Pediatric Telerehabilitation Across Clinical Disciplines

**DOI:** 10.5195/ijt.2024.6684

**Published:** 2025-01-15

**Authors:** Meaghan Reitzel, Lori Letts, Cynthia Lennon, Jennifer Lasenby-Lessard, Monika Novak-Pavlic, Briano Di Rezze, Michelle Phoenix

**Affiliations:** 1 School of Rehabilitation Science, Faculty of Health Sciences, McMaster University, Hamilton, Ontario, Canada; 2 CanChild Centre for Childhood Disability Research, McMaster University, Hamilton, Ontario Canada; 3 KidsAbility Centre for Child Development, Waterloo, Ontario, Canada; 4 Parent partner, Waterloo, Ontario, Canada; 5 Psychology Department, University of Guelph, Guelph, Ontario, Canada; 6 Bloorview Research Institute, Holland Bloorview Kids Rehabilitation Hospital, Toronto, Ontario, Canada

**Keywords:** Caregiver experience, Clinician experience, Multidisciplinary, Pediatric telerehabilitation

## Abstract

**Scope:**

This study describes the high and low points of caregiver and clinician experiences with pediatric telerehabilitation with consideration for the sustainable adoption of pediatric telerehabilitation beyond the COVID-19 pandemic context.

**Methods:**

As part of a larger study, this project analyzed data from qualitative interviews to describe caregivers’ (n = 27) and clinicians’ (n = 27) experiences with pediatric telerehabilitation.

**Findings:**

Caregiver and clinician experiences with pediatric telerehabilitation are described according to four touchpoints identified: (1) child engagement in telerehabilitation; (2) perceived value of telerehabilitation services and caregiver engagement; (3) preparing the people and environment for telerehabilitation services; (4) fit of using a telerehabilitation model; and (5) providing family with choice.

**Discussion:**

Findings highlight the importance of being informed about the telerehabilitation service model, feeling prepared for telerehabilitation appointments and being responsive to families’ choice. Recommendations to address these areas are discussed.

Telerehabilitation, therapy occurring remotely over a telecommunications platform (Parmanto & Saptono, 2009), was rapidly implemented as a model of service delivery because of restrictions on in-person appointments during the COVID-19 pandemic. In the field of pediatric rehabilitation, it is estimated that the number of clinicians using telerehabilitation to provide services grew from 4% to 75% during the COVID-19 pandemic (Camden & Silva, 2021). Although the quick adoption of pediatric telerehabilitation is closely linked to factors related to the COVID-19 pandemic, it has been recommended that the benefits of continuing to offer telerehabilitation alongside in-person appointments as part of a hybrid approach to pediatric rehabilitation be explored (Camden & Silva, 2021; Rosenbaum et al., 2021). Prior to the COVID-19 pandemic, publicly-funded pediatric rehabilitation appointments at Children's Treatment Centres in Ontario, Canada primarily occurred in-person. However, since telerehabilitation was integrated into service provision in response to pandemic restrictions, telerehabilitation continues to be available as an option for families in a hybrid model of practice. These services can include a combination of occupational therapy (OT), physiotherapy (PT), and speech and language pathology (SLP), along with other supportive services such as social work (SW) or behavioural supports.

A 2023, systematic review examining the effectiveness of telerehabilitation interventions compared to other interventions (i.e., no treatment, usual care and in-person) found that on average telerehabilitation interventions were more effective (for 46.9% of outcomes) or as effective (for 53.1% of outcomes), at improving therapeutic outcomes related to the parent and child (Ogourtsova et al., 2023). The effectiveness of pediatric telerehabilitation is further supported by a 2020 systematic review reporting that 56.1% of the outcomes evaluated improved with telerehabilitation intervention (Camden et al., 2020). Telerehabilitation interventions were found to be most effective when they supported implementation of an exercise program by the parent, targeted the parent (i.e., not working directly with the child) and when a coaching approach was utilized (Camden et al., 2020). Alongside literature discussing the effectiveness of pediatric telerehabilitation, preliminary evidence suggests that pediatric telerehabilitation is feasible within the clinical context and acceptable to caregivers (Tanner et al., 2020). The findings from a 2022 scoping review exploring the acceptability of telerehabilitation interventions provided by pediatric occupational therapists and physical therapists suggest that further research is needed to understand how acceptability is defined and evaluated in relation to telerehabilitation intervention (Dostie et al., 2022).

A body of literature has emerged that explores the perspectives of invested groups (e.g., caregivers, youths, and clinicians) about pediatric telerehabilitation. Experience of providing or receiving pediatric rehabilitation services has highlighted both benefits and challenges associated with this service model. Challenges identified include difficulties using the technology, a lack of access to the technology required for appointments, privacy concerns, and distractions in the surrounding environment (Lindsay et al., 2023). Increased flexibility, convenience, and opportunities for the child to be in their own home have been identified as some of the benefits of pediatric telerehabilitation (Lindsay et al., 2023).

At this time, much of the pediatric telerehabilitation literature explores the perspectives of invested groups in isolation of each other (i.e., either clinician or caregiver perspective) (Fairweather et al., 2021; Grant et al., 2022; Wittmeier et al., 2022) or explores the perspectives of multiple invested groups but only related to a single clinical discipline (i.e., caregiver and clinician perspectives related to telerehabilitation with SLP) (Kwok et al., 2022a). Additionally, the literature exploring the perspectives of invested groups as well as the benefits and challenges of pediatric telerehabilitation is often situated in the timeframe of the COVID-19 pandemic when implementation and adoption was rapid and essentially mandatory (Kwok et al., 2022a; Lindsay et al., 2023; Wittmeier et al., 2022).

There is a paucity of evidence describing the experiences of both caregivers and clinicians with pediatric telerehabilitation in a post-pandemic context that is inclusive of multiple clinical disciplines. This study aims to address this knowledge gap by answering the research question: How do caregivers and clinicians describe the high and low points of their experiences with pediatric telerehabilitation? The aim of reporting these findings is to identify potential priorities for change to pediatric telerehabilitation that would enhance caregiver and clinician experiences with this service model. This study was part of a larger Experience Based Co-Design (EBCD) project, with an overall purpose of improving experiences with telerehabilitation services at a publicly-funded children's treatment centre (Reitzel et al., 2023). Implications for the sustainable adoption of pediatric telerehabilitation beyond the COVID-19 pandemic context are discussed.

## Methods

### Study Design

EBCD uses collaborative methods to learn about the experiences of service users (e.g., caregivers) and service providers (e.g., clinicians and health service managers) to guide co-designed changes to health services (Bate & Robert, 2007; Moll et al., 2020; Mulvale et al., 2019). Guided by the EBCD methods proposed by Bate and Robert (2007) the following stages were completed as part of the co-design project: (1) setting up the project; (2) engaging clinicians and gathering their experiences; (3) engaging families and gathering their experiences; (4) co-designing meetings; (5) sustaining co-design engagement and implement change; (6) celebrating and evaluating changes to health service.

This paper focuses on reporting findings related to the experiences of caregivers and clinicians collected during stages 2 and 3 of the co-design process. The aim of stages 2 and 3 is to gain a deep understanding of individuals’ experiences engaging with a health service to uncover collective touchpoints, representing the highs and lows of engaging with a health service such as pediatric telerehabilitation, and to identify priority areas for change (Bate & Robert, 2007; Donetto et al., 2015). To learn more about the full EBCD study and related co-developed solutions refer to the paper by Reitzel et al. (2023). Ethical approval for this study was received by the Hamilton Integrated Research and Ethics Board (project #14235).

### Context

Thorough description of the study context improves trustworthiness of qualitative research by enhancing readers’ understanding of the transferability to other contexts (Lincoln & Guba, 1986). This study was completed in partnership with KidsAbility, a publicly-funded CTC in Southwestern Ontario, Canada. At the time of this project, KidsAbility had six clinical sites providing family-centered therapy services to children from birth to secondary school exit in both urban and rural areas. Prior to the COVID-19 pandemic, most appointments at KidsAbility took place in-person. However, since the pandemic, telerehabilitation visits are offered alongside in-person appointments as part of a hybrid service delivery model. A research partnership was formed with KidsAbility in part due to author MR's history working as an occupational therapist with this organization. Aligning with the collaborative methods of EBCD, our research team, referred to in EBCD as the steering committee, guided each stage of this project and included diverse perspectives from multidisciplinary researchers, clinicians and a caregiver.

### Recruitment

Recruitment for this phase of the project launched in September 2022 and closed December 2022. Caregivers with children who received telerehabilitation services from KidsAbility in the previous 12 months were recruited by self-referral. Established communication channels between KidsAbility and families such as KidsAbility's social media platforms, website and email were used to reach out to caregivers. Clinicians with experience providing telerehabilitation services at KidsAbility in the last 12 months also self-referred and were recruited through messages to their workplace emails and advertising in the internal staff newsletter. A time frame of 12 months was selected for both caregivers and clinicians to ensure that their experiences were representative of the current status of telerehabilitation service provision and not of that provided in mandatory response to the COVID-19 pandemic in March 2020. To be included in the study, clinicians were required to be actively working for KidsAbility in one of the following disciplines: board certified behaviour analyst (BCBA), instructor therapist (IT), communicative disorders assistant (CDA), occupational therapist (OT), physiotherapist (PT), speech-language pathologist (SLP) or social worker (SW). Throughout this paper the term clinician can refer to an individual from any of these clinical disciplines.

The aim was to recruit approximately 30 caregivers and 30 clinicians to maximize variation according to child's age, discipline of service, site of service, urban-rural geography, and caregivers’ gender (Kuzel, 1999). Maximum variation sampling was selected in response to recommendations from KidsAbility's Parent Advisory Committee (PAC) emphasizing the importance of having a diverse sample representative of the services and families connected with KidsAbility. Consultation with the PAC was held prior to commencing recruitment planning. Transportation and language interpretation services were made available in all phases of this project to enhance the accessibility of participation.

### Data Collection and Analysis

Semi-structured interviews were completed with 27 caregivers and 27 clinicians between October 2022 and December 2022. The interviews ranged in length from 20 minutes to 60 minutes. One interview was completed with the assistance of an interpreter. Interviews were completed virtually using the Zoom platform and audio recorded using the laptop microphone (Zoom Video Communications, 2023). The option for in-person interviews was made available to enhance accessibility but was not utilized by any participants. Author MR completed interviews with caregivers, while author MNP interviewed clinician participants to avoid discomfort and to aid in mitigating power imbalances related to author MR interviewing her clinical colleagues. The interview guide was developed by the steering committee with input from members bringing a caregiver and clinical perspective. The same interview guide was used for caregiver and clinician interviews. During the interview, questions were posed to elicit stories from participants about their experiences receiving or providing telerehabilitation services with KidsAbility.

The aim of this phase of analysis was to uncover touch points, which are memorable highs and lows of engaging in telerehabilitation to identify priorities for change (Bate & Robert, 2007). Data from the interviews were analyzed using inductive qualitative content analysis as described by Elo and Kyngäs (2008). The transcripts were read multiple times, and inductive open coding was used to identify codes that highlighted positive and negative aspects of the telerehabilitation experience as described by the participants. Transcripts were coded in Microsoft (MS) Word (Microsoft Corporation, 2016). Google Jamboard (Google, n.d.) was utilized as a collaborative platform to compare and group codes between caregiver and clinician data into higher order categories (Elo & Kyngäs, 2008). In qualitative content analysis, when data are analyzed at a manifest level, codes and categories are meant to capture meaning as it is explicitly expressed by participants (Lindgren et al., 2020). This level of analysis aligns with the aim of identifying touch points as described by the participants while sharing their experiences with telerehabilitation. To enhance trustworthiness, the emerging codes from five caregiver transcripts and five clinician transcripts were reviewed by two researchers on the team (MNP and MP) to validate the codes and identify possible gaps. Halfway through open coding, a summary of the emergent touch points was reviewed by the caregiver and clinical steering committee members (JLL and CL) to ensure the findings resonated with their experiences and to once again draw attention to any gaps or alternate perspectives that should be considered.

Next, author MR led the steering committee in a journey mapping elicitation activity where using Google Jamboard (Google, n.d.), touch points were mapped onto a timeline representing the journey of a telerehabilitation appointment (i.e., time before the appointment, during the appointment and follow up from the appointment). The journey mapping activity provided a visual depiction of when participants were experiencing the touch points during their telerehabilitation journey and facilitated collaborative discussion among the steering committee to prioritize the touch points that would be carried forward into the stage 4 co-design meetings (Reitzel et al., 2023). Trustworthiness was enhanced during the analytic process through use of an audit trail and analytic memos documenting decisions made by the steering committee and monthly peer debriefing meetings.

## Findings

### Sample

Refer to Table 1 for a detailed description of the sample. Thirty-three caregivers were enrolled and 27 participated in this phase of the study. We did not receive responses to schedule interviews from five caregivers and one was unable to participate due to an unforeseen family circumstance. Using a tool developed in Research Electronic Data Capture (REDCap) (Harris et al., 2019), demographic data were collected about 27 caregivers and 29 unique children. Caregivers were recruited from all six KidsAbility sites, 24 participants were mothers, two were fathers and one was a grandparent. Only one caregiver was interviewed from each household except for one family where both the mother and father were interviewed separately regarding their experience with telerehabilitation services. Most families (n = 24) had one child who received services from KidsAbility, however three families identified having two children who received services from KidsAbility. Twenty-three caregivers identified English as the primary language spoken at home, one spoke Urdu, one spoke Persian, one spoke Arabic and one family identified speaking both Arabic and Kurdish. All caregivers indicated having access to a reliable internet connection at home.

**Table 1:** Description of Caregiver and Clinician Sample

Caregivers		Clinicians	
Total caregiver participants	n = 27 Only one caregiver interviewed from each household except for one family where 2 caregivers were individually interviewed	Total clinician participants	n = 27
Total number of children connected to caregiver participants	n = 29	Clinician reported gender	Woman, n = 26 Man, n = 1
Caregiver role	Mother, n = 24 Father, n = 2 Grandparent, n = 1	Clinical discipline	SLP, n = 10 OT, n = 4 PT, n = 4 SW, n = 4 CDA, n = 2 IT, n = 2 BCBA, n = 1
Primarily language spoken at home	English, n = 23 Arabic, n = 1 Arabic and Kurdish, n = 1 Persian, n = 1 Urdu, n = 1	Clinical experience (years)	1 to 5, n = 12 6 to 10, n = 7 11 to 15, n = 1 16 to 20, n = 3 25 +, n = 4
Age of children receiving services from KidsAbility (years)	0 to 3, n = 15 4 to 7, n = 10 8 to 11, n = 3 12 to 15, n = 0 16 to 18, n = 1	Telerehabilitation experience (years)	0 to 2, n = 19 3 to 5, n = 6 6 to 9, n = 1 10 +, n = 1
Diagnoses	Speech and language delay, n = 18 Autism spectrum disorder, n = 4 Global developmental delay, n = 1 Other, n = 15 Preferred not to share, n = 1 Multiple diagnoses, n = 8		
Telerehabilitation received by clinical discipline	Autism services, n = 3 SLP, n = 24 OT, n = 11 PT, = 2 SW, n = 2		
Format of telerehabilitation	Individual sessions, n = 23 Group sessions, n = 1 Individual and group sessions, n = 3		

The age of the children receiving services from KidsAbility ranged from 0 to 18 years old, however 25 of the 29 children represented in the sample were seven years old or younger. Diagnoses represented include speech and language delay (n = 18), autism spectrum disorder (ASD) (n = 4), global developmental delay (n = 1), and other (n = 15). One family preferred not to disclose the diagnosis and eight families identified that their child had multiple diagnoses. Twenty-four families reported receiving telerehabilitation services from SLP, eleven from OT, three from Autism Services (IT or BCBA), two from PT and two from SW. Eighteen families engaged with one discipline for telerehabilitation appointments, six received telerehabilitation services from two clinical disciplines and three families reported engaging in telerehabilitation appointments with more than two clinical disciplines. Twenty-three families reported that they received telerehabilitation sessions that were completed individually with the caregiver and child, one family indicated they only participated in group telerehabilitation and three families shared that they received a combination of individual and group-based telerehabilitation sessions.

Twenty-nine clinicians were enrolled into the study and 27 interviews were completed. One clinician opted to withdraw from the study and another changed place of employment and therefore was no longer eligible. Most clinicians identified their gender as woman (n = 26) and one as a man. Five of the six CTC sites were represented in the primary work site of the clinician participants. There was no clinician representation from one of the two rural sites. The clinical backgrounds of the participants included SLP (n = 10), OT (n = 4), PT (n =4), SW (n = 4), CDA (n = 2), IT (n = 2), BCBA (n = 1). This meant that the sample included at least one member of each clinical discipline providing telerehabilitation services at KidsAbility. All KidsAbility clinical services programs were represented in the sample of clinicians who participated in this phase of the project, including services for children aged 0–3 years, services for school aged children (aged 4 years to secondary school exit up to 21 years old), autism services, and services with a specialized focus (e.g., augmentative and alternative communication). Twelve clinicians reported having 1 to 5 years of clinical experience, seven reported 6 to 10 years, one reported 11 to 15 years, three reported 16 to 20 years and four reported having over 25 years of clinical experience. Of the participating clinicians, 70 % (n = 19) reported having two or fewer years of experience providing telerehabilitation services as part of their clinical practice.

### Touchpoint Identification

From data analysis, four touchpoints were inductively identified from the stories that caregivers and clinicians shared during interviews about their experiences with telerehabilitation. The four touch points identified were: (1) child engagement in telerehabilitation; (2) perceived value of telerehabilitation services and caregiver engagement; (3) preparing the people and environment for telerehabilitation services; (4) fit of using a telerehabilitation model; and (5) providing family with choice. The findings related to each touch point are presented below, synthesizing the perspectives of the caregivers and clinicians on each touch point, which are summarized in Table 2. Findings related to the codesign process that followed the identification of these touchpoints are reported in Reitzel et al. (2023).

**Table 2:** Summarizing the Highs and Lows of Experiences with Pediatric Telerehabilitation

		Caregivers	Clinicians
Child engagement in telerehabilitation	High	More engaged when activities incorporated toys at home	Became more flexible to adapt activities according to what toys family had at home
	Low	Challenges with keeping the child interested in screen-based activities	More preparation required compared to in - person appointments Desire for continued training
Connection between perceived value of telerehabilitation services and caregiver engagement	High	Opportunity to receive in the moment coaching from clinicians Feeling actively involved in sessions More confident carrying out strategies at home Made progress towards goals	Supportive way to coach families Opportunity to have a discussion to establish shared expectations
	Low	Expected clinician to work directly with child Caregiver feeling they had to be too involved in session	Some difficulties establishing a connection with families Unsure of evidence for effectiveness of telerehabilitation
Preparing the people and environment for telerehabilitation services	High	Allows child to be in an environment they are comfortable in Strategies tailored to home environment to target goals	Observing child in the environments they are comfortable in (e.g., home) Tailoring strategies to the home environment and coaching caregivers on how to implement at home
	Low	Distractions in the home environment Unsure how to set up and prepare for a telerehabilitation appointment Managing multiple things during appointment (e.g., child and technology)	Distractions in the home environment Safety considerations of not being physically present with child and family
Fit of using a telerehabilitation model and providing family with choice	High	Telerehabilitation offers flexibility	Telerehabilitation offers flexibility
	Low	Fit between goal of visit and telerehabilitation Comfort with using technology Not having choice in service model	Fit between goal of visit and telerehabilitation Troubleshooting difficulties with technology Assuming families have access to technology and know how to use it

#### Child Engagement in Telerehabilitation – Caregiver Perspective

Caregivers described that at times it was challenging to support their child's engagement in virtual sessions. “Sometimes he would be very reluctant to participate. There was one time when the clinician was showing him a picture of a snake and he said, ‘I will say snake one time, then we are done.’ and he didn't want to do anything else.” (Caregiver 1) Difficulties with child engagement at times left caregivers questioning the value of the appointment, “I can remember an appointment where it felt very pointless because my son would have ‘NO’ days…He didn't even want to look at the computer screen. Now I don't know if that would have been different in-person. He still might have been ‘no, no, no’, but the fact that he didn't want to sit in front of the computer meant that there was no opportunity to do anything.” (Caregiver 2)

When comparing their child's engagement in a virtual session to an in-person session a caregiver shared, that “[in-person] the therapist made them play the games together and against each other. This is more fun than playing against…the computer remotely.” (Caregiver 3) A child's age was a factor some caregivers felt impacted engagement in telerehabilitation sessions. “He was just too young to engage with Zoom.” (Caregiver 3) From the caregiver perspective, using only screen-based activities was felt to limit children's engagement. “Children are very tactile, and they need to do and experience things for learning. I think having the virtual only and having an activity only on a screen was a big limitation as opposed to if parents were given something tactile that the child could have that corresponded to the virtual session.” (Caregiver 1) Caregivers described that incorporating the child's own toys into the sessions improved engagement levels. “After one or two sessions it was pretty clear that our therapist was okay and encouraged my daughter to get some of her toys to interact with and to do some of the exercises with. I would get the computer set-up and my daughter would run and grab her puzzle or whatever.” (Caregiver 4)

Caregivers emphasized the importance of having high quality telerehabilitation materials to support child engagement, “I think I would have liked the little book game and some of the other games that he played with, that were haphazardly put together, to be better. It was as if they had taken pictures of a book they already used. Those could have been a little bit better.” (Caregiver 2) Sustaining a child's engagement for the duration of a session was described to be challenging at times. “Towards the end of each session you could tell he was fading and wanting to go, so getting him to try and participate was like pulling teeth.” (Caregiver 1) When it felt challenging to engage a child in a telerehabilitation appointment caregivers described sessions feeling “pointless,” which was “disappointing” and “frustrating.” Reducing the length of a telerehabilitation session was a strategy caregivers used to increase their child's engagement. “That [30–45 minutes] is a long time for a three- to four-year-old to sit in front of a screen so I started to ask for [the time] to be reduced so we would get more out of the session, and we did.” (Caregiver 10)

#### Child Engagement in Telerehabilitation – Clinician Perspective

Clinicians discussed feeling that it took more time to prepare for telerehabilitation appointments compared to in-person sessions. Creativity was needed to find and adapt engaging activities for an online environment, which children tired of quickly according to clinicians. “I just found I had to be very creative with looking around for what online games and things were available because I could make a power-point presentation but that got really boring, really fast.” (Clinician 1) “There are some really good online games and things too. There are some good websites. The problem is you only get so far and then you have to start paying. It is really not cost effective to buy all of the things because the kids will only really be interested in them for maybe the next three sessions.” (Clinician 1)

Clinicians emphasized the need to be flexible when planning for and conducting engaging telerehabilitation sessions. “I think I had to learn to be a lot more flexible. I found it took a bit of thinking because…if they were in-centre, I have this toy and I'll grab it off the shelf and I know the child is really going to like this, but mom doesn't have that toy at home, so I need to shift my thinking to ‘What do you have at home?’ and what could we do this with right here and right now on the fly.” (Clinician 2)

Clinicians expressed the desire for ongoing training and the opportunity to learn about what other clinicians were doing in their telerehabilitation sessions to support a child's engagement. “I would be really interested in learning about what other therapists are doing externally or internally virtually because I feel as a newer OT, I spend a lot of time trying to figure out how to provide [telerehabilitation] service…because we do not see what other people are doing. There may be other OTs who are doing something totally different… I think it would be helpful to learn what other virtual sessions look like.” (Clinician 3) “I would love to learn about the green screen and how to make use of it and other technologies.” (Clinician 1) “I think having some of that information on-going and learning more about the research into [telerehabilitation] and what best practices are related to telerehabilitation. I think that would be helpful.” (Clinician 4). Without these ongoing opportunities for learning, clinicians expressed concern that they would become less proficient with delivering telerehabilitation services, “[telerehabilitation] is one of those things that you have to continuously use it, or you get a little bit rusty.” (Clinician 5)

#### Connection between Perceived Value of Telerehabilitation Services and Caregiver Engagement – Caregiver Perspective

Caregiver statements such as, “I don't know if it was just me, but it felt like a waste of time.” (Caregiver 5) indicates a perceived lack of value for the telerehabilitation service model. The level of caregiver engagement required during telerehabilitation sessions did not always align with caregiver expectations. “I felt like the virtual was more the speech pathologist teaching me and doing things with me that I could do with [my son] versus trying to address him and giving him words and telling him what to do.” (Caregiver 6) “I felt [telerehabilitation] forced me to be too involved.” (Caregiver 7) A mismatch in caregiver engagement expectations appeared to impact caregivers’ satisfaction and their perceived value of the service they received. A caregiver stated, “I felt like a lot of the onus was put on me to work with my daughter as opposed to my expectation, which was the therapist would be doing the teaching with my daughter and not teaching me to teach my daughter. I was frustrated by that sort of thing.” (Caregiver 4) “Am I the one in speech therapy or is he in speech therapy?” (Caregiver 6)

The need for caregiver engagement in telerehabilitation sessions was not a concern to all caregivers interviewed, some caregivers viewed their increased active engagement as a benefit. One caregiver shared, “I think I put more effort in being actively involved virtually. I do not know why. I think I would have maybe been a little bit more passive and more of just a supervisor [in-person] as opposed to when I was virtual. I was actively trying to be more engaged in listening and being able to carry on the things [the clinician] was doing in the meetings.” (Caregiver 7) Caregivers recalled accounts of their active engagement in telerehabilitation sessions, “[The clinician] would walk me through the things I could do with my daughter in forming the words and what not. She would teach me the touch cues so I could do that on my end with my daughter” (Caregiver 8). In-the-moment coaching and feedback during telerehabilitation sessions was valued by some caregivers. “The second part that stood out was the immediate feedback. Everybody likes a compliment, but I do not know how I am doing [with implementing strategies] sometimes until I see the specialist at the next session…I have not really had anybody until then just come out on their own and say, you are doing a good job.” (Caregiver 9) Caregivers’ perceived value of telerehabilitation services may have been higher if they felt their child was making progress towards their goals. “Our speech therapist has been absolutely amazing, and I see the improvement in my daughter with her speech and so does everyone else. She is improving all the time, so I do not think there is any real downfall for her doing it online.” (Caregiver 8)

#### Connection between Perceived Value of Telerehabilitation Services and Caregiver Engagement – Clinician Perspective

The perceived value that some clinicians placed on telerehabilitation was related to things like developing therapeutic connection and evidence for this service model. Some clinicians with experience providing both in-person and telerehabilitation services identified challenges with developing a therapeutic connection with families during online appointments. “Maybe it is just me, but I feel there is a limitation of the connection and rapport that you build with families… It could just be the way I build relationships, but I feel there is a trust, a closeness and a rapport that does not look the same in virtual therapy as it does when you are face-to-face. You see the body language, but you do not get all of it. You are missing part of the context.” (Clinician 5) Clinicians desired access to evidence about the benefits and limitations of telerehabilitation services to share this information with caregivers and feel validated in offering this service model. “I think the other piece again would be to have some level of validation that it is still providing the same type of change as in-person.” (Clinician 5) “The research of the benefits of virtual services so we can share that with families and say, the research is showing us that children or families make the same gains or skills in-person and virtually, and these are the benefits. At least that way, we are not doing things because we have to but because clinically it makes sense.” (Clinician 1)

Caregiver buy-in was identified as an important foundation for telerehabilitation appointments, “The other thing I think was huge was buy in…If the family didn't buy into the fact that…we could be successful virtually. Having families not understand its value or not accepting what could be provided virtually.” (Clinician 6) A shared understanding between caregiver and clinician about what to expect from telerehabilitation services was described as an important foundation to successful sessions: “I think what makes up a strong interaction is having an understanding of what is expected. So, having talked to the family that the child is not expected to sit at the computer, or what the session is going to be, that it is typically helpful when parents come prepared with questions, things that they have worked on and are wondering about.” (Clinician 3). Caregiver trust was another factor that clinicians felt impacted caregivers’ perceived value of telerehabilitation and engagement. “The families who were really hesitant about [telerehabilitation], they questioned the validity or benefits of it to begin with…but they had to trust us a little bit. I think a lot of time families do not necessarily trust that [telerehabilitation] is going to be a good approach, but I think that often goes along with services in general because often families come in with a preconceived notion that we are going to work directly with their kids, and they are going to be sitting in the corner not doing anything. They are very surprised when we tell them to come to the floor and play with us.” (Clinician 2) Telerehabilitation was viewed as a service model with potential to increase parent engagement in therapy sessions. “A big thing that we push, especially in our programs, is around that building parent capacity piece and that the parent is the one to be carrying out treatment at home. I think because there is such a focus on that coaching model, especially with certain types of skills, I think that a virtual model is really a nice supportive way to coach families and have a conversation with them.” (Clinician 7)

#### Preparing the People and Environment for Telerehabilitation Services – Caregiver Perspective

Caregivers described not knowing what to expect when getting started with telerehabilitation appointments. “Early days it is like your first baby. You do not have a clue. To have that guidance of ‘this is how therapy works’ and ‘this is how we are going to move forward’. Now I know what the plan is and what we are doing, but those early days I really needed a lot of instruction about what our visit was going to look like.” (Caregiver 11) Additionally, caregivers lacked clarity about what their role would be during a telerehabilitation appointment. “Perhaps I went into it with the wrong expectation that I was just going to be there to ensure that my daughter is doing what she is supposed to be doing as opposed to actually being really involved in the situation. That might be helpful for the parent to know what the expectation is and what they can expect and what these virtual sessions will look like.” (Caregiver 9).

Caregivers described feeling like there was a lot for them to manage during the telerehabilitation appointments related to the environment, sharing information with the clinician, and supporting their child's participation. “So here I am trying to hold the iPad and hold my daughter to support her and then it was okay, put the iPad down on the floor at the bottom of the stairs and okay, but [the clinician] was not really getting the full picture of what is going on because the iPad is just not positioned right and you could only see so far.” (Caregiver 8) Another caregiver shared, “It was a lot because I had a non-speaking toddler, so I was doing a lot of the speaking and describing… Then I would also hand him things. Occupational therapy wanted to see if he would take two cars and crash them…For physiotherapy I was literally walking him around or giving him a walker. A lot of times I was running behind him with the computer to try and get a view of him walking. It was very hands on, and I was usually sweating by the end of it.” (Caregiver 2) Caregivers described a tension between wanting to play naturally with their child in the home environment while also ensuring that the clinician could see what was occurring on the screen. “I only had so much space that my camera provides. I would be in the middle of doing something with him and he would move slightly, I would feel that I needed to move my camera to ensure that the therapist could see what was going on and see if what I am doing is right…But then it would interrupt the whole natural interaction that I was having with my son to get him to do that because I would say, ‘oh one second, Mommy has to grab the iPad’, and at that point the moment was lost.” (Caregiver 12)

Caregivers shared that at times, distractions in the home environment made telerehabilitation appointments challenging. “We did [telerehabilitation] almost exclusively while the baby was napping to not contend with the baby too often, but there are other distractions around, even their toys, the dog, snacks, and whatever is going on around them.” (Caregiver 13) Despite some challenges, caregivers identified benefits of telerehabilitation appointments occurring in the home environment, such as the child being more comfortable in a familiar environment or tailored strategies from the therapist on how to use their surroundings to target goals. “She was a lot more comfortable with the surroundings she knows. The toys, the blankets and anything we were doing, she would feel more comfortable trying things because she was in the comfort of her own home.” (Caregiver 5) A caregiver shared, “[Our clinician] asked me to go raid my pantry for…his favourite snacks… She had me put pieces of snacks all the way along the couch and at the beginning he reached as far as he could and then took a sidestep. That was the first step movement that he had taken. That was within a [telerehabilitation] session we had gotten him to take a step movement, so that one was huge.” (Caregiver 2)

#### Preparing the People and Environment for Telerehabilitation Services – Clinician Perspective

Clinicians also identified distractions as a challenge with telerehabilitation appointments. “Sometimes you can have the other brothers and sisters coming in and interrupting or other people coming in or phone calls happening…It can be quite disruptive if mom is at home and other people are at home. It is not always the most focused and that can be a thing.” (Clinician 8) Difficulties with the technology, such as audio quality were another obstacle clinicians described. “Sometimes the audio was tricky, especially if I was trying to do a speech assessment. I find especially with iPads, depending on the way they had the camera facing, it seemed to be that the microphone would pick up more of the noise behind the iPad than facing forward, so I would hear everything going on around, but I would not be able to hear the child that I needed to hear.” (Clinician 9)

Safety was an additional environmental consideration that clinicians discussed in relation to telerehabilitation appointments. “Another difficulty is I cannot be there to do hands-on stuff. I cannot be there for safety. Things I would be more than willing to try in-centre to see if we could then use that strategy for home, you cannot. I cannot teach mom enough safety techniques for her body to be safe, or me to figure out how the [child] is going to respond to be able to transfer that skill over to mom with my words.” (Clinician 8)

Similar to caregivers, clinicians also identified benefits of telerehabilitation linked to being able to see the child in their home environment. “I have had some lovely sessions that have involved eating and mealtime, because I think that virtual offers an opportunity to see the home environment that we do not get to see in-person. Sessions where I have noticed things where I might not have identified like positioning during eating or a lot of distractions in the home environment that I may not have been aware of that are affecting eating.” (Clinician 3) “Being able to see home set-ups…to walk through an exercise program and say, ‘I want you to hold onto the counter behind you’. Just being able to use their real-life things instead of having them on the site…I can get them to take me around and show what they are doing.” (Clinician 8) “I have had some nice experiences where families will have the video set-up in the living room where there are toys or if the child has a playroom maybe. That has been good in terms of getting a more representative speech sample of a child because they are in their own space, with their own toys and they are likely to be more relaxed and more likely be able to demonstrate their abilities because they are at home.” (Clinician 10)

A clinician shared that seeing a family for a telerehabilitation appointment gave them “a picture of what their life is like. It can be an eye-opener about how chaotic their lives are, and I am asking them to do more. So, it gives me the perspective of ‘you have a very busy life, how can I help you incorporate our goals that we set together in a realistic way?’” (Clinician 11)

#### Fit of Using a Telerehabilitation Model and Providing Family with Choice – Caregiver Perspective

The importance of the fit of the telerehabilitation model with families’ skills, resources and preferences were described by caregivers. A caregiver felt it was important that clinicians understood a family's familiarity with the technology needed for virtual appointments, noting that there could be cultural aspects that influence their comfort with using it. “I think it is really important to know their culture too. In my experience, we did not have enough experience with virtual. We learned it in Canada. We had to do everything over the phone, and we have never applied for anything online in Iran.” (Caregiver 14).

Caregivers described alignment between the fit of telerehabilitation and their family needs when considering things like time saved travelling to the centre for appointments, eliminating barriers related to transportation and childcare for other children. “A big plus especially at KidsAbility for a parent in my situation where I do not drive and have two kids, so transportation is an issue along with childcare. If I have to, I'll hop on the bus with two kids but trust me, it is not easy when you have a baby whom I cannot leave at home as I have no childcare, so if I show up in-person and my husband is not there, it is very hard for me to focus.” (Caregiver 9) “Virtually or remotely is more efficient because we don't…waste time moving to some other place.” (Caregiver 3) A caregiver also discussed telerehabilitation appointments as a potential protective factor for parents experiencing mental health concerns. “There are days when I say ‘yes, let's do this’ …and when the day comes, I just don't want to do it. I don't want to go out… So having the option for Zoom means that I am not going to cancel those appointments last minute that are going to be beneficial for my son just because I am feeling that I do not want to step outside right now and I cannot handle people.” (Caregiver 15)

Caregivers described that it was important to consider the fit of a telerehabilitation appointment with the type (e.g., assessment) and purpose of the visit. “With a virtual assessment they cannot check for tightness or mobility. They cannot check all of those things that with her specific issue they need to check for. They were trying to get me to manipulate her foot a certain way and ask me if it feels tight, but I do not know. I am an educated person, but I cannot tell you whether a calf muscle is tight or not.” (Caregiver 13)

The importance of giving families choice in the service model they feel will be a fit for them was a critical point emphasized by caregivers. “I think going forward it would be valuable to offer both as options [in-person and telerehabilitation] and not require one or the other. I think if the parents are more willing to be involved, it is more successful for the children. The parents must be active participants in order for the kids to be successful. It is asking parents how can we make this easy for you? Do you want to come in-person, or would you rather do it virtually?” (Caregiver 7)

#### Considering the Fit of Using a Telerehabilitation Model and Providing Family with Choice – Clinician Perspective

When thinking about the fit of telerehabilitation for a family, clinicians highlighted the importance of considering the family's access to the technology required to engage. “A huge limitation is assuming that people have access to the technology and the connectivity to actually do these.” (Clinician 8) Familiarity with using the technology was also discussed by clinicians. “Trying to talk people through how to do [telerehabilitation] if they are not very familiar with technology…that type of thing, it is tricky.” (Clinician 12)

In addition to ensuring families had access to technology and could get connected to the visit, clinicians also identified the importance of considering the fit between a telerehabilitation approach and the goal of the visit. Telerehabilitation was identified as more difficult when a child had goals related to something physical such as fine motor skills. “I think where things got a little more challenging is when you had a child who had more physical needs and it was hard to do a full physical or fine motor assessment without really seeing them.” (Clinician 7) Practicing in a hybrid model offering in-person and telerehabilitation was highlighted by clinicians as beneficial. “The hybrid model has been a really nice thing to do…I like doing the in-person where you are trying to figure out strategies, but once you figure out the strategies, following up virtually has been a really good thing for us.” (Clinician 8)

Clinicians felt they could be more flexible with families when telerehabilitation was incorporated into their treatment plan. Instead of cancelling an appointment clinicians shared that “we can quickly switch over to Zoom. That was a real benefit.” (Clinician 5). The importance of giving family choice of service delivery models was emphasized by clinicians. “[Telerehabilitation] is always an option, and I always ask families what they would prefer.” (Clinician 10) “I do think it is important to provide [families] ultimately with the choice.” (Clinician 3)

## Discussion

The findings describe the experiences of caregivers and clinicians engaging in pediatric telerehabilitation. The stories of their experiences emphasize both high and low points. There is an opportunity to explore how caregiver and clinician experiences differ as well as how they align and to consider their influence on the sustained adoption of telerehabilitation as part of a hybrid approach to pediatric rehabilitation services. The experiences of caregivers and clinicians highlight the importance of being informed about the telerehabilitation service model, feeling prepared for telerehabilitation appointments, and needing to be responsive to families’ choices related to service.

A desire to be informed about telerehabilitation services was highlighted in the narratives of both caregivers and clinicians. However, the type of information desired differed between these groups. Clinicians identified wanting to be informed about the evidence related to the effectiveness of telerehabilitation. However, constraints such as limited time and experience with searching for and appraising literature, prohibits access to empirical evidence that is ready for use in clinical practice (Chan et al., 2010). It is recommended that clinicians and health service organizations make use of widely available evidence-based knowledge translation resources such as the TelereHUB-CHILD (https://telerehubchild.com/) (Ogourtsova, 2023) or CanChild Telepractice resources (https://www.canchild.ca/en/resources/356-telepractice-resources) (Kwok et al., 2022b) that synthesize the evidence related to pediatric telerehabilitation assessment and intervention.

Caregivers shared experiences of not knowing what to expect when joining a telerehabilitation appointment or what their role would be. These experiences echo what is written in the literature about caregivers’ expectations of therapy, where parents describe not having enough information to know what to expect when engaging with therapeutic services and bringing with them a predetermined idea of what their involvement in a session will look like (Phoenix et al., 2020). This gap in knowledge is of critical importance because a shared understanding of expectations for therapy between caregiver and clinician has been shown to increase caregiver engagement in services (King et al., 2019). Clinician narratives indicate that taking time to discuss the telerehabilitation service model and the role of the caregiver in these sessions results in more successful integration of this service model into care. Therefore, it is imperative that caregivers receive information (e.g., knowledge sharing dialogue between clinician and caregiver) about the telerehabilitation service model and their role in these sessions for them to feel informed and enter the appointments with expectations aligned with what the model of service offers. To enhance experiences with telerehabilitation, findings from the co-design work that drew from this interview data recommended that organizations have a process in place to consistently discuss the telerehabilitation service model and caregiver role in telerehabilitation sessions prior to commencing with virtual services (Reitzel et al., 2023).

Having a thorough understanding about the telerehabilitation service model and having access to related evidence is crucial information for determining whether telerehabilitation would be an appropriate fit for a child's therapy plan. Once the determination is made to proceed with telerehabilitation, training is needed for the clinician and caregiver to ensure that they feel prepared to engage in these appointments (Retamal-Walter et al., 2022). This includes familiarity with the required technology; knowledge of how to set-up the environment; and knowing how to adapt therapeutic activities to optimize engagement in telerehabilitation. Clinicians desired opportunities for ongoing training and collaboration with colleagues to foster continued skill development and confidence in providing telerehabilitation. A 2021 systematic review, examining the effectiveness of implementation approaches to support the uptake of evidence-informed interventions in allied healthcare reported higher levels of success with implementation efforts that use multiple strategies (Goorts et al., 2021). The use of opinion leaders (i.e., colleagues with telerehabilitation experience), workshops, and ongoing training are identified as strategies supporting intervention implementation (Goorts et al., 2021). Perceptions of being inadequately trained decrease the likelihood that clinicians will offer telerehabilitation as an option for therapy (Graham et al., 2023). It is recommended that pediatric health service organizations offering telerehabilitation consider utilizing these types of implementation strategies to provide clinicians with opportunities for ongoing skill development and training to enhance their experience with providing telerehabilitation services.

From interviews with caregivers, a key aspect to a caregiver feeling prepared for a telerehabilitation appointment was knowing how to set up the technology and the environment. This information should be reviewed with caregivers ahead of commencing with an appointment to ensure they feel ready to engage. Text-based resources, such as those available through the TelereHUB-CHILD (Ogourtsova, 2023) or CanChild (Kwok et al., 2022b) are available to support clinicians in training caregivers to prepare for telerehabilitation appointments. Alternately, video resources demonstrating how to set up for a telerehabilitation appointment were recommended out of the larger co-design process connected to this project as a method of sharing this information with caregivers (Reitzel et al., 2023). A 2023 evaluation of telerehabilitation with children with neurodevelopmental conditions, reports that early failures and challenges were viewed by clinicians and caregivers as a normal part of the learning process (Graham et al., 2023). Training is needed to support learning in these early phases of telerehabilitation to build knowledge, skills and confidence to persist with using this service model.

Family-centred service necessitates that caregivers have choice in what therapy services for their child look like and that their voices are heard as partners in therapy alongside the clinician (McCarthy & Guerin, 2022; Rosenbaum et al., 1998). When families make the choice to participate in telerehabilitation, their perceived value of the service increases and the transition to incorporating telerehabilitation into care is more successful (Graham et al., 2023). Conversely, telerehabilitation was perceived by caregivers as less valuable when they felt they did not have a choice about whether to engage with this model of service (Graham et al., 2023). When working with children with communication difficulties, providing family-centred telerehabilitation was identified as an important factor for engagement (Retamal-Walter et al., 2023). To be responsive to caregivers’ choices, clinicians need to engage caregivers in shared decision-making regarding service models. Clinicians need to feel confident working in a hybrid model, shifting with relative ease between in-person and telerehabilitation visits.

Although this work incorporated a diverse range of perspectives (i.e., caregiver, clinicians, management, researchers) at each stage of the project, a limitation of this work is that it was conducted with participants from a single clinical organization, potentially limiting the transferability of the findings. We acknowledge that although set in the context of pediatric rehabilitation, the experiences of children and youth with telerehabilitation are not represented in this work and should be incorporated in future research in this area. Additionally, future research should explore how telerehabilitation is being used when incorporated into a hybrid service model along with caregiver and clinician satisfaction with a hybrid approach to pediatric rehabilitation services.

## Conclusion

The interviews conducted with caregivers and clinicians as part of a larger co-design project bring to attention both positive and negative aspects of their experiences with pediatric telerehabilitation. Experience with telerehabilitation is enhanced when caregivers and clinicians have the knowledge and skills to prepare and engage in a telerehabilitation appointment. Furthermore, the perceived value of telerehabilitation increases when caregivers are empowered to make a choice regarding the fit of this service model for their child and family.
